# Timing of depressive episode onset relative to chronic pain reveals distinct patterns of affective and multisite pain

**DOI:** 10.3389/fpsyt.2026.1813589

**Published:** 2026-09-22

**Authors:** Eva Gontrum, Karishma Shah, Ellie Han, Margaret Legarreta, Erin McGlade, James Yancey, Perry Renshaw, Deborah Yurgelun-Todd

**Affiliations:** 1 Diagnostic Neuroimaging, University of Utah, Salt Lake City, UT, United States; 2 Department of Psychiatry, School of Medicine, University of Utah, Salt Lake City, UT, United States; 3 Rocky Mountain Mental Illness Research, Education, and Clinical Center (MIRECC), Salt Lake City Veteran Affairs, Salt Lake City, UT, United States

**Keywords:** affective pain, chronic pain, chronological comorbidity, depression, multisite pain

## Abstract

**Introduction:**

Prior research shows the relationship between chronic pain and depression is bidirectional, with co-occurrence associated with worse functional outcomes. However, few studies have examined the temporal sequence of first depressive episode to the onset of multisite pain. We examined the relationship between timeline of first depressive episode, pain intensity dimensions, and multisite pain.

**Methods:**

Participants were 35 adults reporting chronic musculoskeletal pain for >3 months (54.3% female, age= 35.6 ± 7.2 years, pain duration= 11.3 ± 7.6). Participants were classified into three groups based on the timing of their first depressive episode: first depressive episode prior to chronic pain onset (pre-CP DEP), first depressive episode after chronic pain onset (post-CP DEP), or no history of depressive episode (no DEP). Depressive episode onset was assessed through SCID-IV interviews, and affective pain intensity and multisite pain were measured using the McGill Pain Questionnaire and Lubeck Pain Questionnaire, respectively. Mediation analysis assessed the role of affective pain in the relationship between pre-CP depressive episode and multisite pain.

**Results:**

Pre-CP depressive episode was associated with higher total pain intensity (B = 11.31, 95% CI [0.19, 22.43], *p* = 0.047), affective pain (B = 3.11, 95% CI [0.19, 6.02], *p* = 0.037), and miscellaneous pain scores (B = 3.06, 95% CI [0.02, 6.10], *p* = 0.049) compared to participants without depressive episode history. Pre-CP depressive episode was also associated with increased multisite pain (B = 6.85, 95% CI [0.90, 12.79], *p* = 0.025), with the pre-CP group reporting an average 6.85 more pain sites than the no DEP group. Mediation analysis identified an indirect effect of pre-CP depressive episode whereby affective pain accounted for approximately 43% of the total association between pre-CP depressive episode and number of pain sites.

**Discussion:**

Results suggest that depressive episode prior to onset of chronic pain is associated with greater pain intensity and multisite pain. We provide hypothesis-generating evidence that affective pain may represent an intermediate process linking pre-CP depressive episode to multisite pain, suggesting that premorbid depressive episode may be associated with specific vulnerabilities to both qualitative and spatial characteristics of subsequent chronic pain.

## 1 Introduction

Chronic pain, defined as pain lasting beyond three months and past normal tissue healing time, affects 1 in 5 adults, and is one of the most significant public health concerns in the U.S (1, 2). Strong associations have emerged between chronic pain and depression; up to 60% of chronic pain patients experience comorbid depression, compared to approximately 9% prevalence in the U.S. adult population (3, 4). The relationship between chronic pain and depression is bidirectional; having either disorder increases the risk of developing the other. Chronic pain and depression have also been found to share psychosocial risk factors such as pain catastrophizing, psychological distress, perceived injustice, insomnia, fatigue, and social isolation (5, 6). There is also substantial overlap in biological markers for chronic pain and depression including genetic overlap (7, 8), shared dysfunction of neurotransmission and pro-inflammatory markers (9, 10), and similar disruption of neural circuitry (11). Moreover, comorbid chronic pain and depression are associated with worse clinical prognosis and increased disability compared to either condition alone (12–14). Treatment of chronic pain can ameliorate depression, and conversely, effective management of depressive symptoms often improves pain outcomes (15, 16). Despite robust evidence of comorbidity and shared mechanisms, the temporal dynamic processes through which chronic pain and depressive symptoms interact remain poorly understood. Much of the existing research examines comorbid depression as a binary variable (present or absent) or examines it as concurrently occurring with chronic pain without temporal specificity, limiting our understanding of causal or moderating pathways of the affective components of chronic pain. Understanding when depressive symptoms arise relative to pain onset and progression is crucial, as different temporal patterns may reflect distinct underlying mechanisms and clinical presentations.

One current issue in the field is that chronic pain is generally treated as a unitary construct, without differentiating its sensory and affective components (17). This simplification undermines clinically meaningful variation in pain experience and potentially specific pathways linking pain to depression. The IMMPACT core outcome domains for designing chronic pain clinical trials distinguish between these dimensions. Sensory components of pain refer to the qualities and characteristics of the pain sensation itself (i.e., intensity, duration, and quality), whereas affective components of pain refer to the emotional or distressing aspect of the pain experience (i.e., how unpleasant or distressing it feels) (18). These distinct aspects of self-reported pain may also have different neurological substrates. As pain becomes chronic, neural activity progressively shifts from primary somatosensory regions toward affective and limbic brain areas—including the medial prefrontal cortex, anterior cingulate cortex, and amygdala (19). This shift parallels increases in behavioral withdrawal and avoidance, highlighting the affective dimension of pain as a critical factor in the development and maintenance of chronic pain (20). Furthermore, greater pain intensity is associated with increased psychopathological symptoms and maladaptive coping strategies (21), further underscoring the link between pain intensity and affective components of pain.

Chronic multisite pain, which is inclusive of but not as narrowly defined as chronic widespread pain, is distinct from pain in a single area of the body. Chronic multisite pain is more likely to reflect nociplastic pain than a transient injury or tissue damage, and is often treatment-refractory (22, 23). Nociplastic pain is defined as regional, multifocal, or widespread pain that cannot entirely be explained by nociceptive or neuropathic mechanisms and arises from altered function and increased sensitivity of pain-related sensory pathways in the peripheral or central nervous system (24). Pain can spread across body sites, and when it does, it is associated with increased pain intensity and pain duration (25, 26). The development of multisite pain in adults can be predicted by systemic factors such as maladaptive body awareness, comorbid chronic disease, mood disorders, and current pain. Additionally, sleep problems, nonpainful somatic symptoms, attentional issues, and current pain have been shown to predict new multisite pain in children (27–31). Specifically, psychiatric comorbidities are among the strongest predictors of persistent, disabling multisite pain (32–34). Evidence shows that patients with depressive disorders have more somatic/pain symptoms than controls, and conversely, that the risk of mood disorder increases in proportion to the number of sites of chronic pain reported (8, 23). Population surveys from 17 countries show that diffuse pain was strongly associated with mood and anxiety disorders throughout the world, suggesting that these relationships hold true across cultures (35).

To date, relatively few studies have untangled factors influencing the temporal relationship between mood disorders and chronic multisite pain. Longitudinal research is imperative to understand the temporal dynamics of chronic pain and its comorbid psychiatric disorders. Prior research has shown that early-onset psychiatric disorders, particularly depression, are more strongly associated with subsequent chronic back and neck pain than later-onset psychiatric disorders (12). One study of fibromyalgia similarly distinguishes clinical profiles based on whether depression and fibromyalgia occur concurrently or sequentially (36). In individuals with a sequential pattern of onset, panic disorder most frequently occurs first, followed by major depressive episodes (including bipolar spectrum disorders) and then fibromyalgia (36). Mood and anxiety disorders among adolescents most frequently preceded the onset of fibromyalgia-related chronic pain in clinical settings (37). Together, these findings suggest that for a subset of individuals, environmental and psychiatric conditions causing emotional and physiological arousal prior to age 18 may predispose individuals to early onset anxiety or panic disorder, increasing risk for subsequent multisite pain.

Altogether, the existing literature highlights the importance of understanding multidimensional, temporal models of chronic pain. Chronic pain is closely associated with affective and limbic processes, which significantly modulate the development, maintenance, and intensity of chronic pain. Evidence further suggests that affective processes may not only co-occur with chronic pain but, in some individuals, may precede and influence its development. Disentangling how sensory and affective dimensions of pain interact with affective disorders over time is therefore critical for understanding the causal pathways of chronic pain and informing stage-specific treatment interventions.

In this study, we investigate how the timing of depressive episode onset (before vs. after chronic pain) differentiates between distinct possible chronic pain phenotypes. Very few SCID-based studies of chronic pain exist, although the few that do demonstrate the prevalent psychiatric comorbidity of patients with chronic pain (38, 39). Using the Structured Clinical Interview for DSM-IV (SCID-IV) to establish clinician-validated diagnoses and precise mood disorder onset timing, the current study examines whether the timing of depressive episodes results in distinct chronic pain phenotypes that differ in affective pain intensity and the number of pain sites.

## 2 Materials and methods

### 2.1 Participants and demographics

Participants included 35 adults who reported chronic musculoskeletal pain for > 3 months (54.3% female, mean age = 35.6 ± 7.2, mean pain duration = 11.3 ± 7.6). Participants were recruited through collaboration with local pain clinics and advertising in local communities. Study inclusion criteria included being between 18–50 years of age and reporting musculoskeletal pain for three months or longer as verified in an in-person medical history interview. We excluded individuals with cancer pain, diabetic neuropathy, poststroke pain, or multiple sclerosis. Other exclusion criteria included any neurological disorder diagnosis, history of seizure or severe head trauma, active or history of severe mental illness (i.e., active suicidal ideation or psychosis), substance abuse or dependence in the past three months, or unstable medical conditions. Participants were allowed to continue their medications for pain and other medical conditions as prescribed. Study procedures were approved by the Institutional Review Board at the University of Utah in Salt Lake City, UT.

This study is cross-sectional; all participant data was collected at one study visit. Demographic data collected included age, sex, ethnicity, highest level of education completed, employment status, and relationship status.

### 2.2 Pain questionnaires

Participants completed the McGill Pain Questionnaire, which is designed to examine the subjective qualities of pain (40, 41). The questionnaire consists of a body drawing for individuals to mark their pain locations. Multisite pain was defined as the sum of all marked pain sites, and a map of possible pain sites (maximum 38) can be seen in **Figure 1**. The questionnaire also includes a scale of 80 words that describe the sensory, affective, evaluative, and miscellaneous components of pain. Based on the types of words chosen in each category, total pain intensity is reported from 0-78 (sensory component ranges from 0-42, affective component ranges from 0-14, evaluative component ranges from 0-5, miscellaneous score ranges from 0-17). Participants also completed the Lubeck Pain Questionnaire, which allowed participants to self-identify locations of chronic pain and specify the timeline for when pain started in each location (42, 43).

**Figure 1:**
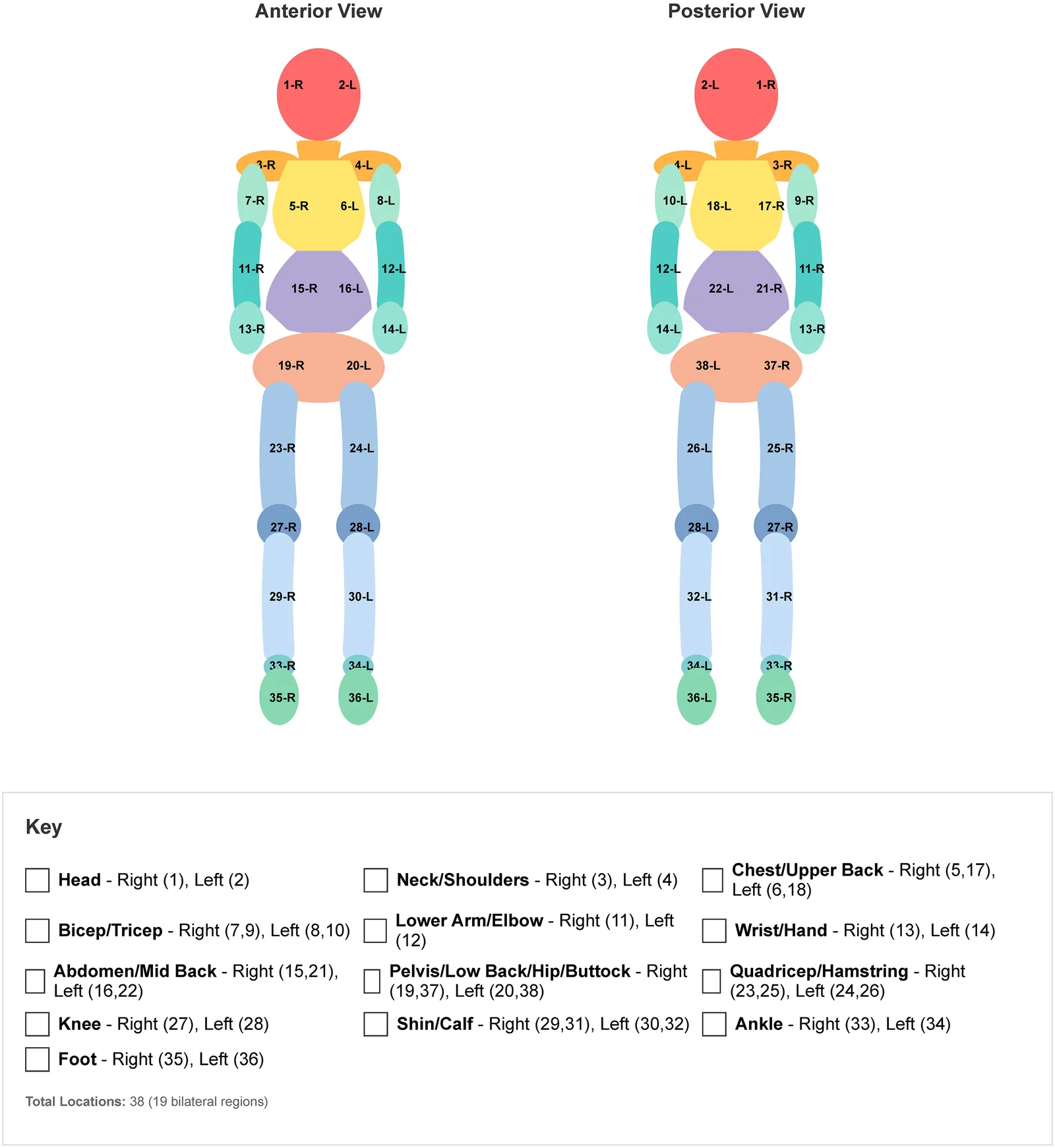
McGill pain questionnaire: pain locations body map.

### 2.3 Structured clinical interview for DSM-IV

Participants underwent a comprehensive psychiatric interview to determine current and past psychiatric diagnoses using the Structured Clinical Interview for the Diagnostic and Statistical Manual of Mental Disorders, Fourth Edition (SCID-IV). The structured interview was conducted face-to-face by a licensed psychologist or supervised postdoctoral psychologist resident. The following diagnoses (current and lifetime) were registered: major depressive disorder (MDD), bipolar disorder, dysthymic disorder, panic disorder, posttraumatic stress disorder (PTSD), generalized anxiety disorder, and substance use disorder (including alcohol and drug abuse and dependence). Lifetime history of depressive episodes was recorded for all participants, regardless of whether criteria were met for a mood disorder. Participants were asked when symptoms of depressive episodes first occurred, and this timeline was compared with the time estimate of pain onset in the pain questionnaire. The timeline comparison allowed participants to be separated into three groups: a pre-CP depressive episode group (n=14, reported a clinical depressive episode before chronic pain onset), a post-CP depressive episode group (n=9, reported a clinical depressive episode after chronic pain onset), and a no depressive episode group (n=12, no history of a clinical depressive episode).

### 2.4 Statistical analysis

All statistical analyses were performed using RStudio version 2025.9.1.401 (Posit team, Boston, MA, USA) and JMP Student Edition 18 (SAS Institute Inc., Cary, NC, USA).

#### 2.4.1 Linear regression analyses

To examine the effects of the timeline of depressive episode onset on pain quality, separate linear regression models were conducted for the McGill Pain Questionnaire Total Pain Rating Index and each McGill Pain Questionnaire subscale (Sensory, Affective, Evaluative, and Miscellaneous). All analyses were performed with cross-sectional data. Depressive episode timeline was entered as a categorical predictor with three levels. Two reference group configurations were examined (1) comparing pre-CP depressive episode and post-CP depressive episode groups to those with no depression history, and (2) comparing pre-CP depressive episode to post-CP depressive episode. Standardized regression coefficients (β) were calculated to facilitate comparison of effect sizes across outcomes with different scales. Unstandardized coefficients (*B*), standard errors, *t*-values, *p*-values, and 95% confidence intervals were reported for all estimates.

#### 2.4.2 Mediation analysis

We conducted an exploratory mediation analysis to assess whether the association between pre-CP depressive episode and the number of sites of pain was consistent with an indirect pathway through affective pain score. Although all variables were measured cross-sectionally, we used a causal mediation framework given the inherent temporal ordering associated with our depressive episode variable. This timeline comparison between depressive episode(s) and chronic pain was based on the earliest reported depressive episode and the earliest reported site of chronic pain. Causal interpretations are contingent on the assumed temporal ordering (pre-CP depressive episode → affective pain score → number of sites of pain).

Three ordinary least squares regression models were estimated to characterize the component paths of the mediation model:

1. Mediator model (path a)

 • Predictor → Mediator: Affective pain score was regressed onto pre-CP depressive episode.

2. Outcome model (paths b and c’)

 • Mediator and Predictor → Outcome: Number of pain sites was regressed onto pre-CP depressive episode and affective pain score.

3. Total Effect model (path c)

 • Predictor → Outcome: Number of pain sites was regressed onto pre-CP depressive episode.

Using the fitted regression models, we estimated the average causal mediation effect (ACME), the average direct effect (ADE), the total effect, and the proportion mediated. Confidence intervals and p-values were derived using a quasi-Bayesian Monte Carlo approach with 5,000 simulations. Analyses were performed in R software using RStudio (version 2025.9.1.401) using the ‘mediation’ package.

A sensitivity analysis was conducted to assess the robustness of the estimated indirect effect to potential bias introduced by unmeasured confounding of the mediator-outcome relationship (44). This analysis used the ‘medsens’ function (45) to examine how strong unmeasured confounding between the mediator and outcome would need to be to reduce the indirect effect to zero. This was quantified using the correlation between error terms (ρ) and the proportion of variance explained by an unmeasured confounder (product R^2^ metrics).

## 3 Results

### 3.1 Participant characteristics

Participants (n = 35) were classified into three groups based on the timing of their first depressive episode: first depressive episode occurred prior to chronic pain onset (pre-CP DEP, n = 14, 40.0%), first depressive episode occurred after chronic pain onset (post-CP DEP, n= 9, 25.7%), or no history of depressive episode (no DEP, n = 12, 34.3%) (**Table 1**). Comparative analyses revealed no significant demographic group differences. However, group differences in age neared significance (p=0.06), so *post-hoc* comparisons were performed. Significant group differences in age emerged only between the pre-CP and post-CP DEP groups (p = 0.05).

**Table 1:** Demographic and clinical characteristics by depression timeline.

Characteristic	Whole sample (N = 35)	Pre-CP depressive episode (n = 14, 40.0%)	Post-CP depressive episode (n = 9, 25.7%)	No depressive episode (n = 12, 34.3%)	P-value
Age in years	35.60 ± 7.19	38.21 ± 7.27	31.00 ± 6.67	36.00 ± 6.24	0.06
Female sex	19 (54.3)	10 (71.4)	5 (55.6)	4 (33.3)	0.15
Racial or ethnic identity					
Asian or Pacific Islander	1 (2.9)	1 (7.1)	0 (0.0)	0 (0.0)	
Latino/Hispanic	2 (5.7)	0 (0.0)	1 (11.1)	1 (8.3)	
White	29 (82.9)	12 (85.7)	7 (77.8)	10 (83.3)	0.24
More than one Race	3 (8.6)	1 (7.1)	1 (11.1)	1 (8.3)	
Education (years)	14.11 ± 2.10	14.21 ± 2.26	13.67 ± 1.41	14.33 ± 2.42	0.75
Pain Duration (years)	11.30 ± 7.64	9.32 ± 7.17	11.00 ± 6.36	13.83 ± 8.83	0.33

CP, Chronic pain. Data presented as mean ± standard deviation or n (% based on available data) as appropriate. For comparative group analyses, one-way ANOVAs were performed for continuous variables, and Chi-Square tests of independence were performed for categorical variables.

Based on SCID DSM-IV criteria, 21 participants met criteria for a lifetime mood disorder and 12 met criteria for a current mood disorder (**Table 2**). Major depressive disorder was the most prevalent diagnosis, with 19 participants meeting criteria for past MDD, and 9 meeting criteria for current MDD. Anxiety disorders were also prevalent across groups, with 10 participants meeting criteria for current and past generalized anxiety disorder. There were 12 participants with a history of PTSD, 7 with a history of alcohol abuse or dependence, and 10 with a history of other drug abuse or dependence. Other than mood and anxiety disorders, lifetime psychiatric disorders were well-distributed across groups as assessed with Chi-square tests of independence. Significant group differences were noted with mood disorders (p<0.001) and anxiety disorders (p=0.004). This is to be expected as we grouped participants based on depressive episode history, and anxiety disorders tend to co-occur with mood disorders (46–48).

**Table 2:** Current and past psychiatric diagnoses based on SCID DSM-IV criteria.

Diagnosis	Whole sample (n = 35)		Pre-CP depressive episode (n = 14)		Post-CP depressive episode (n = 9)		No depressive episode (n = 12)		P-value
	Past	Current	Past	Current	Past	Current	Past	Current	Lifetime
Any mood disorder	21	12	13	7	8	4	0	1	<0.001**
MDD	19	9	12	6	7	3	0	0	
Dysthymic disorder	1	1	0	0	0	0	1	1	
Bipolar disorder	2	2	1	1	1	1	0	0	
Any anxiety disorder	13	10	7	5	4	3	2	2	0.004**
GAD	10	10	5	5	3	3	2	2	
Panic disorder	3	0	2	0	1	0	0	0	
PTSD	12	6	5	3	3	1	4	2	0.99
Alcohol abuse or dependence	7	0	2	0	3	0	2	0	0.50
Other drug abuse or dependence	10	0	3	0	2	0	5	0	0.23

CP, Chronic Pain; MDD, Major Depressive Disorder; GAD, Generalized Anxiety Disorder; PTSD, Posttraumatic Stress Disorder. Other drug abuse or dependence is inclusive of the following substances: cannabis, amphetamines, cocaine, and opioids. Comparative group analyses were performed using Chi-Square tests of independence. For group analyses, we used lifetime diagnoses and did not distinguish between past and current. **p<0.01 indicates statistical significance.

### 3.2 Effects of depression timeline on McGill pain questionnaire subscales

Linear regression analyses revealed that pre-CP depressive episode is associated with higher scores on PRI-Total (B = 11.31, 95% CI [0.19, 22.43], *p* = 0.047), PRI-Affective (B = 3.11, 95% CI [0.19, 6.02], *p* = 0.037), and PRI-Miscellaneous (B = 3.06, 95% CI [0.02, 6.10], *p* = 0.049) compared to participants with no history of depressive episode (**Table 3**). *Post hoc* comparisons demonstrated that participants with a post-CP depressive episode did not differ significantly from either pre-CP or no depressive episode groups on all pain measures (**Table 3**). However, we note that participants with post-CP depressive episode consistently showed intermediate pain scores that fell between the other groups (**Figure 2**).

**Table 3:** Linear regression coefficients for timeline of first depressive episode predicting McGill pain questionnaire subscales.

Variables	*B*	S.E.	95% CI	*t*	*p*
PRI-total					
*Reference: No DEP*					
Intercept (No DEP)	29.833	4.007	[21.67, 37.99]	7.446	<0.001**
Pre-CP DEP	11.310	5.460	[0.19, 22.43]	2.071	0.047*
Post-CP DEP	6.944	6.121	[-5.52, 19.41]	1.135	0.265
*Reference: Pre-CP DEP*					
Post-CP DEP	-4.365	5.930	[-16.44, 7.71]	-0.736	0.467
PRI-sensory					
*Reference: No DEP*					
Intercept (No DEP)	19.667	2.142	[15.30, 24.03]	9.179	<0.001**
Pre-CP DEP	4.333	2.920	[-1.61, 10.28]	1.484	0.148
Post-CP DEP	2.333	3.273	[-4.33, 9.00]	0.713	0.481
*Reference: Pre-CP DEP*					
Post-CP DEP	-2.000	3.171	[-8.46, 4.46]	-0.631	0.533
PRI-affective					
*Reference: No DEP*					
Intercept (No DEP)	3.250	1.050	[1.11, 5.39]	3.095	0.004**
Pre-CP DEP	3.107	1.431	[0.19, 6.02]	2.171	0.037*
Post-CP DEP	1.083	1.604	[-2.18, 4.35]	0.675	0.504
*Reference: Pre-CP DEP*					
Post-CP DEP	-2.024	1.554	[-5.19, 1.14]	-1.302	0.202
PRI-evaluative					
*Reference: No DEP*					
Intercept (No DEP)	2.333	0.412	[1.49, 3.17]	5.660	<0.001**
Pre-CP DEP	0.810	0.562	[-0.33, 1.95]	1.441	0.159
Post-CP DEP	0.778	0.630	[-0.51, 2.06]	1.235	0.226
*Reference: Pre-CP DEP*					
Post-CP DEP	-0.032	0.610	[-1.27, 1.21]	-0.052	0.959
PRI-miscellaneous					
*Reference: No DEP*					
Intercept (No DEP)	4.583	1.095	[2.35, 6.81]	4.187	<0.001**
Pre-CP DEP	3.060	1.492	[0.02, 6.10]	2.051	0.049*
Post-CP DEP	2.750	1.672	[-0.66, 6.16]	1.645	0.110
*Reference: Pre-CP DEP*					
Post-CP DEP	-0.310	1.620	[-3.61, 2.99]	-0.191	0.850

*B*, unstandardized coefficient; S.E., standard error of the coefficient; *β*, standardized coefficients; 95% CI, confidence interval for the unstandardized estimates (B); PRI, Pain Rating Index. DEP = Depressive Episode. N = 35 (Pre-CP DEP n = 14, Post-CP DEP n = 9, No History of DEP n = 12). Confidence intervals for intercepts refer to baseline values. Confidence intervals for variables refer to regression estimates relative to baseline values. For each McGill subscale, coefficients are shown for two reference groups: (1) Reference: No DEP compares Pre-CP and Post-CP DEP to the No DEP group; (2) Reference: Pre-CP DEP compares Post-CP DEP to the Pre-CP DEP group. *p < 0.05 and **p < 0.01 indicate statistical significance.

**Figure 2:**
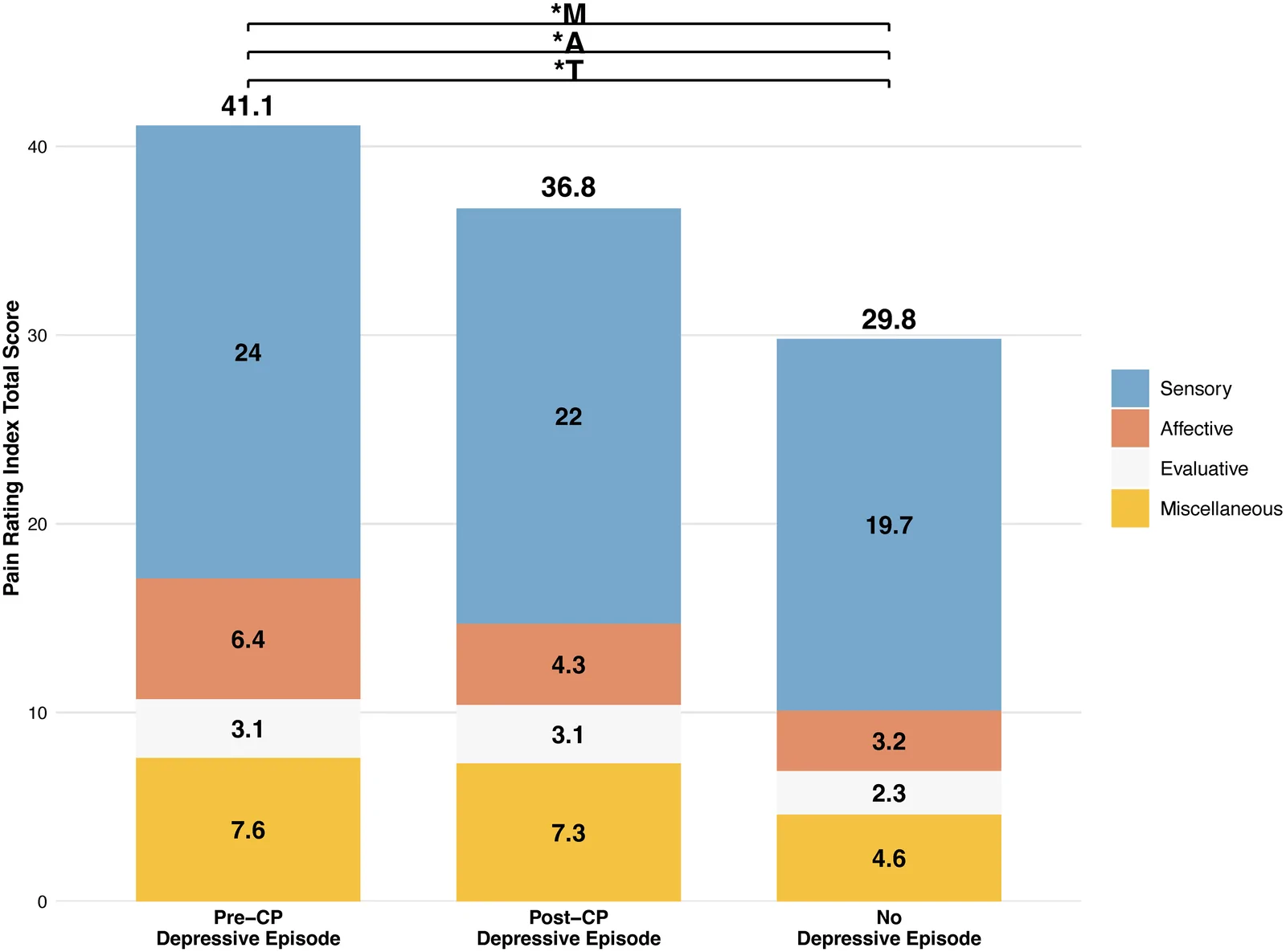
McGill pain rating index subscales by timeline of first depressive episode. Participants with pre-CP depressive episode reported significantly higher PRI- total, affective pain, and miscellaneous pain than participants with no history of a depressive episode. Bars marked with asterisks indicate significant differences for *p< 0.05, *T = Total (p = 0.047), *A = Affective (p = 0.037), and *M =Miscellaneous (p = 0.049).

### 3.3 Effects of timeline of depressive episode on multisite pain

Linear regression models showed that participants who experienced a pre-CP depressive episode reported increased multisite pain when compared to participants with no history of depressive episode (B = 6.85, 95% CI [0.90, 12.79], *p* = 0.025) such that the pre-CP group has, on average, 6.85 more pain sites than the no depressive episode group (**Figure 3**). *Post hoc* comparisons demonstrated that post-CP depressive episode was not associated with a significant difference in sites of pain when compared to pre-CP or no depressive episode groups (**Table 4**).

**Figure 3:**
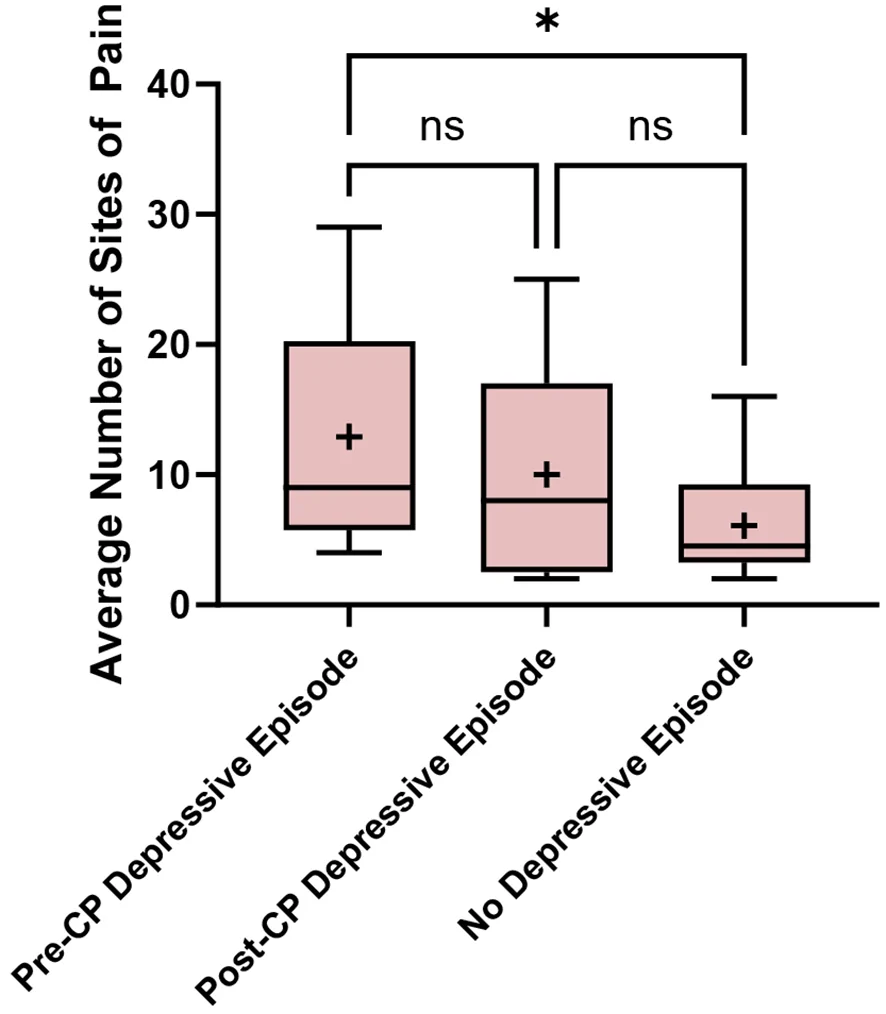
Average number of pain sites by timeline of depressive episode. Individuals with pre-chronic pain depressive episode reported significantly more pain sites (M = 12 ± 8.84), than those with no depressive episode (M = 6.08 ± 4.23, p = 0.022). The box extends from the 25^th^ to 75^th^ percentile. The median is plotted as a line in the middle of the box. The + sign is plotted at the mean. The whiskers extend from the smallest value to the largest. *p<0.05.

**Table 4:** Linear regression coefficients for depressive episode timeline predicting multisite pain distribution.

Variables	B	S.E.	95% CI	*t*	*p*
Multisite pain (all locations)					
*Reference: No DEP*					
Intercept (No DEP)	6.083	2.141	[1.72, 10.44]	2.842	0.007**
Pre-CP DEP	6.845	2.917	[0.90, 12.79]	2.346	0.025*
Post-CP DEP	3.917	3.270	[-2.74, 10.58]	1.198	0.240
*Reference: Pre-CP DEP*					
Post-CP DEP	-2.929	3.168	[-9.38, 3.53]	-0.924	0.362

*B*, unstandardized coefficient; S.E., standard error of the coefficient; *β*, standardized coefficients; 95% CI, confidence interval for the unstandardized estimates (B); N = 35 (Pre-CP DEP n = 14, Post-CP DEP n = 9, No DEP n = 12). Confidence intervals for intercepts refer to baseline values. Confidence intervals for variables refer to regression estimates relative to baseline values. Multisite Pain (All Locations) represents the total number of pain sites reported across all body locations. For the outcome, coefficients are shown for two reference groups: (1) Reference: No DEP compares Pre-CP and Post-CP DEP to the No DEP group; (2) Reference: Pre-CP DEP compares Post-CP DEP to the Pre-CP DEP group. *p < 0.05 and **p < 0.01 indicate statistical significance.

### 3.4 Affective pain mediates the relationship between pre-CP depressive episode and multisite pain

Regression analyses indicated that, in the Mediator model, the effect of pre-CP depressive episode on affective pain score was statistically significant (B = 3.11, 95% CI [0.18, 6.03], *p* = 0.04), with the affective pain score of participants who experienced ≥1 pre-CP depressive episode being 3.11 higher than participants who had never experienced a depressive episode (**Table 5**). In the Outcome model, the effect of the mediator (affective pain score) on the number of pain sites was significant (B = 1.00, 95% CI [0.26, 1.73], *p* = 0.01), but the direct effect of pre-CP depressive episode on the number of pain sites was no longer statistically significant (B = 3.75, 95% CI [-1.84, 9.34], *p* = 0.18). In the Total Effect model, when the number of pain sites was only regressed onto pre-CP depressive episode alone, the total effect was statistically significant (B = 6.85, 95% CI [1.08, 12.61], *p* = 0.02), with participants who experienced ≥1 pre-CP depressive episode reporting 6.85 more pain sites than participants who had never experienced a depressive episode.

**Table 5:** Regression analysis results of pre-CP depressive episode, affective pain, and number of pain sites.

Regression models							
Model	Dependent variable	Independent variable(s)	Estimate (B)	SE	95% CI	*t*	*p*
Mediator model	Affective Pain	(Intercept) No DEP Pre-CP DEP	3.25 3.11	1.04 1.42	[1.10, 5.40] [0.18, 6.03]	3.125 2.192	0.005** 0.04*
Outcome model	Pain sites	(Intercept) No DEP Pre-CP DEP Affective Pain	2.85 3.75 1.00	2.15 2.70 0.36	[-1.59, 7.29] [-1.84, 9.34] [0.26, 1.73]	1.327 1.389 2.804	0.20 0.18 0.01*
Total Effect model	Pain sites	(Intercept) No DEP Pre-CP DEP	6.08 6.85	2.05 2.80	[1.85, 10.32] [1.08, 12.61]	2.966 2.449	0.006** 0.02*

*p < 0.05, **p < 0.01.

The exploratory mediation model showed that the estimated indirect effect of pre-CP depressive episode on the number of pain sites through affective pain score was statistically significant (ACME = 3.14, 95% CI [0.16, 7.57], *p* = 0.04) (**Table 6**). After including affective pain score in the model, the direct effect of pre-CP depressive episode was no longer significant (ADE = 3.71, 95% CI [-1.65, 9.09], *p* = 0.18). The total effect of pre-CP depressive episode on pain sites was significant (Total Effect = 6.85, 95% CI [1.05, 12.90], *p* = 0.02), with the indirect pathway accounting for approximately 43% of the total effect (**Figure 4**).

**Table 6:** The mediating role of affective pain between pre-CP depressive episode and the number of pain sites.

Mediation model				
	Estimate	95% CI	*p*	Sensitivity (ACME = 0)
Indirect Effect (ACME)	3.14	[0.16, 7.57]	0.04*	ρ = 0.50
Direct Effect (ADE)	3.71	[-1.65, 9.09]	0.18	--
Total Effect	6.85	[1.05, 12.90]	0.02*	--
Proportion Mediated	0.44	[-0.001, 1.59]	0.05*	--
Sensitivity (residual variance)	--	--	--	R^2M~^ * R^2Y~^ = 0.12
Sensitivity (product R^2^)	--	--	--	R^2M^ * R^2Y^ = 0.25

ACME, average causal mediation effect; ADE, average direct effect. Sensitivity parameters indicate the magnitude of unmeasured confounding required to reduce the estimated indirect effect to zero. Estimates are based on 5,000 simulations. Sample size = 26. *p<0.05 indicates statistical significance.

**Figure 4:**
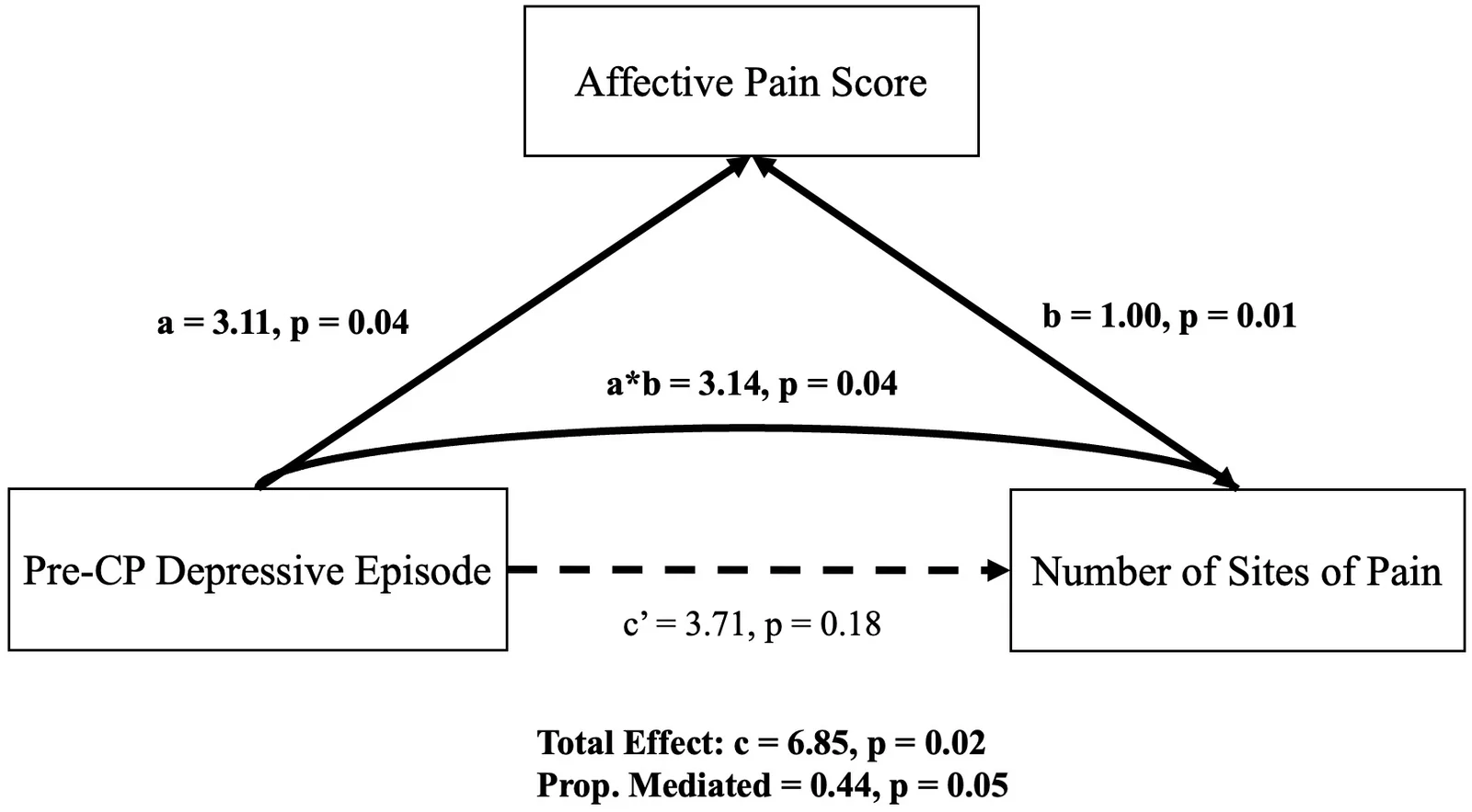
Mediation model showing the indirect effect of pre-chronic-pain depressive episode(s) on the number of sites of pain through affective pain score. Solid arrows indicate statistically significant paths (p<0.05), and dashed arrows indicate non-significant paths. Approximately 44% of the total effect was mediated through the affective pain score. Path **(a)** represents the effect of pre-CP depressive episode on affective pain score (mediator). Path **(b)** represents the effect of affective pain score (mediator) on the number of pain sites. Path (a*b) represents the indirect effect of pre-CP depressive episode on the number of pain sites through affective pain score (mediator). Path (c’) represents the direct effect of pre-CP depressive episode on the number of pain sites after accounting for affective pain score (mediator). The variable **(c)** represents the total effect of pre-CP depressive episode on the number of pain sites without accounting for affective pain score (mediator).

A mediation sensitivity analysis was conducted to evaluate the robustness of the estimated indirect effect to potential unmeasured confounding between the mediator and the outcome. The estimated ACME was reduced to zero only when the correlation between the mediator and outcome error terms exceeded ρ = 0.50 (**Figure 5**). This level of confounding corresponds to an unmeasured variable explaining ~12% of the residual variance in both the mediator and the outcome, or ~25% of their total variance. This analysis suggests that the estimated mediation effect is robust to moderate violations of the sequential ignorability assumption.

**Figure 5:**
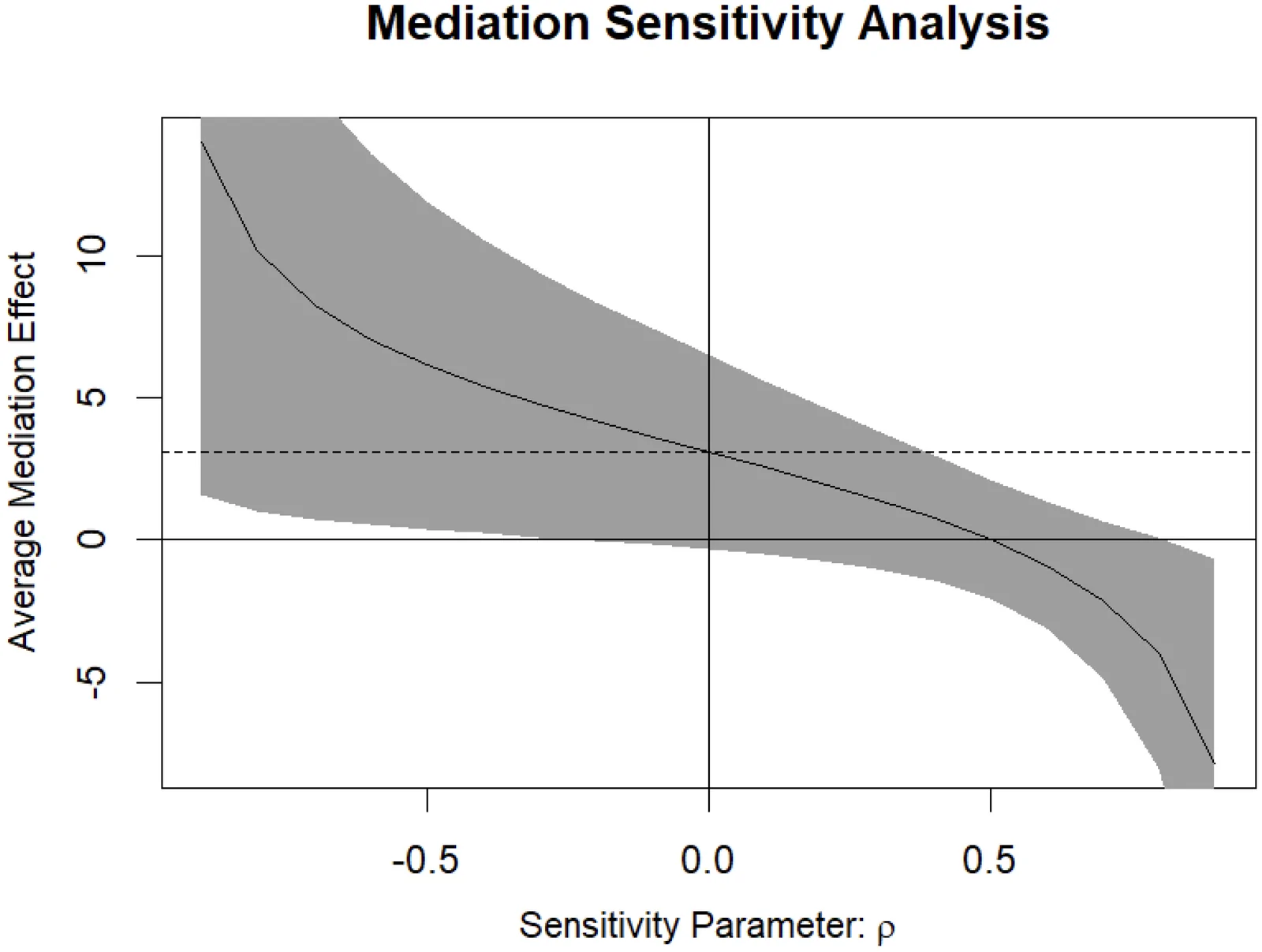
Sensitivity analysis for the confounding assumption. The curve represents the average causal mediation effect (ACME) of affective pain score on the number of pain sites as a function of the sensitivity parameter rho (ρ). The shaded area indicates the 95% confidence interval, the horizontal dotted line denotes the null indirect effect (ACME = 0), and the vertical line at ρ = 0 represents the assumption of no residual correlation between the mediator and outcome models. The value of ρ for which the natural indirect effect is equal to zero is 0.5.

## 4 Discussion

### 4.1 Pre-existing depressive episode is associated with greater pain intensity

We examined how the timing of depressive episodes relative to chronic pain onset distinguishes potentially unique chronic pain phenotypes with different patterns of pain experience and multisite pain. Our exploratory analyses suggest that individuals with a first depressive episode occurring prior to chronic pain onset (pre-CP) demonstrate significantly elevated pain intensity scores, including total pain intensity, affective pain, and miscellaneous pain, compared to those with no history of depressive episode.

Prior work demonstrates that depression and negative affect amplify the affective component of pain, independent of nociceptive intensity (49). The affective component of pain may predict psychological and social disability, while the sensory components are more strongly associated with physical disability (50). Our hypothesis-generating findings complement this literature and align with longitudinal evidence demonstrating the reciprocal relationship between chronic pain and depression. Specifically, baseline depressive symptoms were associated with increased likelihood of subsequent chronic pain, while greater initial pain severity predicts later depressive symptoms (51–54).

One biologically plausible explanatory framework to explain the greater affective pain observed among pre-CP participants is altered affective and limbic pain processing. While our analyses are exploratory, they do allow us to speculate as to the mechanism behind them. Neuroimaging studies indicate that neural activity progressively shifts from primary somatosensory regions, primarily nociceptive, towards affective-emotional and limbic brain regions during the chronification of pain, regions that are also central to mood regulation (19). It has been suggested that a depressive episode occurring prior to pain onset may “prime” affective and limbic circuits, lowering the threshold for affective pain amplification and increasing sensitivity to distressing aspects of pain, which may lead to maladaptive response-focused emotion regulation strategies (20). This hypothesis is supported by experimental evidence showing that controlled induction of negative mood increases pain unpleasantness and limbic reactivity (55). Direct associations have been found between maladaptive response-focused emotional regulation and chronic pain, pain-related disability, and depressive symptoms, suggesting that emotional regulation may be a secondary risk factor for chronic pain (20).

These functional neuroimaging changes mentioned previously likely reflect underlying neurobiological alterations. These include NMDA receptor overactivation in the spinal cord, altered neuropeptides and neurotrophic factors, monoaminergic dysregulation, and excitatory-inhibitory imbalance (56–58). Dysfunction in the descending pain pathways, specifically the serotonergic dorsal raphe nucleus and noradrenergic locus coeruleus, leads to abnormal pain amplification and disrupted emotional regulation (58). Neuropsychiatric clustering is present in many conditions, including chronic pain (59–62). Depression, anxiety, sleep disturbance, and somatic distress tend to cluster and emerge together across conditions, potentially reflecting shared mechanistic pathways of immune dysregulation, neuroinflammation, oxidative stress, and altered central processing (63–65). Recent studies on long COVID and neuropsychiatric manifestations suggest there are neurobiological alterations present in both COVID-19 and psychiatric disorders (64, 66, 67). This raises the possibility that there may be shared affective-neurobiological mechanisms across many conditions, not just chronic pain and depression. Taken together, these findings are consistent with a conceptual model in which affective and somatic symptoms may arise from similar and synergistic mechanistic pathways. However, further analyses with a larger sample size, longitudinal design, and greater ability to adjust for confounding variables are necessary to attribute our findings to any biologic mechanism.

### 4.2 Pre-existing depressive episode is associated with more sites of pain

Pre-CP depressive episodes were associated with substantially more sites of pain, with an average of 6.85 additional pain sites compared to individuals without history of depressive episode. Post-CP individuals exhibited intermediate levels of pain intensity and multisite pain but did not differ significantly from either the pre-CP or no depressive episode groups.

Multisite pain may be more likely to reflect nociplastic mechanisms and also appears to be qualitatively different than single-site pain. Multisite pain differs from single-site pain in that it has disproportionately stronger associations particularly with depression, but also with anxiety, sleep disturbance, and disability than single-site pain, and these associations remain even after adjusting for pain severity (68). This may be partly explained by stronger genetic components (8, 69), reduced hippocampal gray matter volume (14), and greater associations with most inflammatory markers than single-site pain (68). Large-scale neuroimaging studies of chronic pain show that widespread alterations in cortical surface area and thickness were most robustly associated with multisite pain, with precuneus thickness mediating the relationship between chronic multisite pain and history of suicide attempt (70). Multisite pain, more so than single-site pain, may affect central nervous system processes in a more sustained way and may be tied to more psychiatric vulnerability. The close relationship between multisite pain and psychiatric comorbidity provides context for examining how depression might alter pain processing before chronic pain develops.

### 4.3 Affective pain mediates the relationship between pre-CP depressive episode and multisite chronic pain

An exploratory mediation analysis examined whether the association between pre-CP depressive episode and the number of pain sites was consistent with an indirect pathway through the affective pain score. Regression analyses indicated that pre-CP depressive episodes were associated with affective pain scores and number of pain sites, and that affective pain scores were significantly associated with number of pain sites. The mediation analysis built on these associations and identified a statistically significant indirect effect of affective pain score that accounted for ~43% of the total association between pre-CP depressive episode and number of pain sites. While the direct effect of pre-CP depressive episode was not statistically significant after accounting for the mediating effect of affective pain score, the estimated direct effect remained non-zero with a wide confidence interval. Under the assumed causal ordering (pre-CP depressive episode → affective pain score → number of sites of pain), this pattern is consistent with a partial mediation effect. Sensitivity analysis suggested that the estimated indirect effect was relatively robust to moderate violations of the sequential ignorability assumption. Taken together, these findings provide hypothesis-generating evidence that affective pain score may represent an intermediate process linking pre-CP depressive episode to multisite pain. Premorbid depression may specifically amplify the emotional or distressing aspects of the pain experience rather than merely increasing sensory intensity. While previous studies have focused primarily on the co-occurrence and mutual contribution of the two conditions and their intensity, our findings highlight that affective and multisite pain may be important features of chronic pain in individuals with a history of premorbid depressive episode.

The exploratory mediation model is supported by the neurobiological alterations that occur during heightened affective pain described above, which may help explain how depression is connected to multisite pain. Although causal mechanisms remain incompletely described, our preliminary findings support existing hypotheses that premorbid depressive episodes may reduce central pain inhibitory capacity and allow for amplification of pain and spatial spreading across body regions (56, 71, 72). It has been reported that depression moderates endogenous pain modulatory processes, with reduced pain inhibitory capacity being particularly associated with pain intensity in participants with higher depression severity (73). Our hypothesis-generating findings suggest that a premorbid depressive episode might confer specific vulnerabilities to both qualitative and spatial characteristics of subsequent chronic pain.

Furthering understanding of how affective processes may precede and influence the development of chronic pain may have diagnostic and treatment implications. Individuals with depressive episodes prior to chronic pain may benefit from interventions specifically targeting affective pain components and emotional regulation. For example, recent studies have shown that vortioxetine, a newer antidepressant that modulates serotonergic neurotransmission, can relieve both depressive and pain symptoms simultaneously (74). Additionally, a study of lifestyle factors suggests that there may be some benefit to treating comorbid chronic pain and depression as a distinct condition with a unique profile of associated risk factors, including primarily loneliness and insufficient sleep (75). Ultimately, treatments that simultaneously target the affective and somatic dimensions of chronic pain may offer greater therapeutic benefit by addressing overlapping neurobiological mechanisms.

### 4.4 Limitations and future directions

One of the limitations of this study is that it is a cross-sectional study and the exploratory results need to be verified in a prospective study. The mediation analysis used cross-sectional variables, limiting the model’s ability to establish temporal precedence or to speak to definitive causality. The mediation model also relies on the assumption of sequential ignorability, and, while the sensitivity analysis suggests robustness to violations of this assumption, unmeasured confounding variables remain possible. Furthermore, the sample size for the mediation model (n=26) limits statistical power and the precision of estimates, as evidenced by relatively wide confidence intervals. Due to these limitations, the findings from this mediation analysis remain exploratory and should be interpreted cautiously as an investigation of associational relationships rather than establishing causality. Additionally, the timeline variables derived from the SCID interviews that allowed us to create a temporal ordering of depression rely on retrospective recall, which introduces the possibility of recall bias. Future studies should employ a longitudinal design and prospective randomization to establish causality in these relationships. Furthermore, the current study does not capture how intraindividual pain is variable across time, and future studies may benefit from longitudinal models that include dynamic changes in pain and depressive symptoms (76).

A second limitation of the current study is the modest sample size of the individual groups, which limits the statistical power and interpretability of findings. However, given the paucity of temporal data collected for both psychiatric symptoms and pain variables, this pilot study provides important preliminary data. Additionally, given our modest sample size, our models did not control for age, sex, comorbid psychiatric disorders, or concurrent use of pain or psychiatric medications, all of which could contribute to the observed associations. Based on these limitations, the findings should be considered preliminary and the analyses should be considered hypothesis-generating. Future studies should explore sex-dependent influences on these associations, given that prior work has noted potential sex-based differences in depression and chronic pain (77). Age-related factors are similarly known to influence both chronic pain and depression (78, 79). The co-occurrence of anxiety disorders, PTSD, and substance disorders in this sample may also contribute to both affective pain and multisite pain burden (80–82). Approximately 50% of our sample reported taking any kind of pain medication and 40% of the cohort reported taking a psychiatric medication. While medication administration may have impacted the pain scoring, we propose that it was less likely to affect the timing of diagnosis.

Another challenge in the interpretation of the current data is that the use of multisite pain, by definition, adds complexity and variability to our findings. Many studies restrict their samples to specific pain conditions, such as low back pain, musculoskeletal pain, neuropathic pain, or migraine. By contrast, our inclusion of diverse pain presentations sacrifices some discriminatory specificity in favor of capturing the broader clinical reality of multisite pain and increasing the generalizability of our findings. All participants endorsed chronic musculoskeletal pain in some capacity, and the multisite pain variable was meant to capture pain that might co-occur with musculoskeletal pain, even if from different etiologies. Additionally, pain conditions frequently co-occur and share common genetic and neurobiological mechanisms (83–85).

The current study also has several important strengths, including use of the SCID administered by clinical psychologists to establish a detailed timeline of clinician-validated diagnoses, rather than relying on self-report questionnaires. Complete psychiatric histories allow for determination of mood disorder onset timing relative to chronic pain onset, which is relatively uncommon in the literature. We specifically distinguish depressive episodes occurring before versus after chronic pain onset, and differentiate between multiple components of pain, rather than treating pain as a single construct. Additionally, including multisite pain in our analyses better captures the clinical reality of chronic pain presentations, improving external validity compared to studies restricted to single pain conditions. Despite a modest sample size, the mediation analysis with sensitivity testing provides initial hypothesis-generating evidence for a mechanistic pathway.

## 5 Conclusions

To our knowledge, this is the first study to examine chronic pain presentations based on whether depressive episodes precede or follow pain onset, linking these temporal patterns to distinct affective and multisite pain profiles. These preliminary results extend our current understanding of the reciprocal relationship between depression and chronic pain by suggesting that when a depressive episode precedes chronic pain onset, it is associated with greater pain intensity, particularly affective pain, and greater multisite pain. Our hypothesis-generating results posit that depressive episodes preceding chronic pain onset may introduce specific vulnerabilities to developing the widespread, treatment-refractory pain phenotype characteristic of nociplastic conditions. These initial results emphasize the importance of developing a multidimensional and temporal model of chronic pain that accounts for when psychiatric conditions emerge relative to pain onset with future longitudinal data.

## Data Availability

The raw data supporting the conclusions of this article will be made available by the authors, without undue reservation.
